# Efficacy and Safety of Dexmedetomidine as an Adjuvant to Bupivacaine in Ultrasound-Guided Pericapsular Nerve Group (PENG) Block for Proximal Femoral Fractures: A Randomised, Single-Centre Controlled Trial

**DOI:** 10.7759/cureus.102853

**Published:** 2026-02-02

**Authors:** Ruchi Singh, Nazia Nazir, Anupriya Saxena, Samiksha Khanooja, Ankit Kataria, Akshay Panwar, Vikas Saxena

**Affiliations:** 1 Anesthesiology and Critical Care, Government Institution of Medical Sciences, Greater Noida, IND; 2 Orthopaedics, Government Institution of Medical Sciences, Greater Noida, IND

**Keywords:** bupivacaine, dexmedetomidine, nerve block, pericapsular nerve group (peng), postoperative pain, proximal femur fracture, randomized controlled trial

## Abstract

Background: Dexmedetomidine has been used in many peripheral nerve blocks, as it enhances the duration of analgesia and hastens its onset. We compared whether adding dexmedetomidine to bupivacaine in the pericapsular nerve group (PENG) block resulted in prolonged duration of analgesia with ease of positioning for spinal anaesthesia in proximal femur fractures without adverse effects.

Methods: This prospective randomised study was conducted in a tertiary care hospital in North India between January 2024 and June 2024 after institutional ethics approval and Clinical Trials Registry of India (CTRI) registration (CTRI/2024/12/078728). Sixty adult patients aged 18-60 years, with American Society of Anesthesiologists (ASA) physical status I-III, presenting with proximal femur fractures less than seven days old and moderate to severe pain (numeric rating score (NRS) > 4), scheduled for surgery, were randomly allocated to Group A or Group B. Group A received 20 mL of 0.25% bupivacaine with 50 mcg dexmedetomidine (0.5 mL) for the PENG block, while Group B received 20 mL of 0.25% bupivacaine with 0.5 mL of saline for the PENG block. The primary outcome was the duration of analgesia. Secondary outcomes included ease of spinal positioning score, postoperative pain scores, postoperative opioid requirement, quadriceps strength at 24 hours, and adverse events.

Results: In this single-centre trial, the use of dexmedetomidine as a perineural adjuvant in the PENG block was associated with a prolonged duration of analgesia (95% CI: 2.89-4.71; p < 0.001), better positioning scores (p < 0.001), and lower postoperative pain scores compared to bupivacaine alone in 60 patients aged 18-60 years. Motor function was preserved in both groups, and no significant adverse events were observed.

Conclusions: Dexmedetomidine is a safe and effective adjuvant for the PENG block in proximal femur fracture surgeries in adults aged 18-60 years. Further studies in geriatric populations are required for broader clinical recommendations.

## Introduction

Hip fractures are emerging as a major global health problem, with an estimated 1.6 million cases occurring worldwide annually [1]. Proximal femur fractures include fractures involving the neck of femur, pertrochanteric and subtrochanteric regions. These fractures are associated with delayed postoperative mobilisation, which results in increased complications, prolonged hospital stays, and higher morbidity [2]. Regional anaesthesia techniques have revolutionised the perioperative management of proximal femur fracture surgeries by providing opioid-sparing analgesia and facilitating early mobilisation [3].

Femoral nerve block has been the gold standard for analgesia for proximal femur fracture surgeries for many decades. However, due to quadriceps weakness, postoperative mobilisation has been delayed. The fascia iliaca compartment block emerged as a safer alternative, but inadequate analgesia of the hip capsule resulted due to innervation from deeper and medial articular branches. Hence, pericapsular nerve group (PENG) block was proposed as a motor-sparing block that selectively targets the articular branches of the anterior hip capsule, facilitating early recovery after hip surgeries [4].

Dexmedetomidine is a highly selective alpha-2 adrenergic agonist that has been extensively studied as an adjuvant to local anaesthetics in various regional anaesthetic techniques. It is known to improve the duration and onset of analgesia as an adjuvant [5,6]. Despite its established role as an adjuvant in peripheral nerve blocks, there is a paucity of evidence supporting the use of dexmedetomidine as an adjuvant to bupivacaine in ultrasound-guided PENG block.

The primary objective of this study was to compare the duration of analgesia following ultrasound-guided PENG block using dexmedetomidine as an adjuvant to bupivacaine versus bupivacaine alone in proximal femur fractures. Secondary objectives included assessment of ease of spinal positioning (EOSP), postoperative pain scores in the first 24-hour period, 24-hour opioid consumption, quadriceps muscle strength at 24 hours postoperatively, and adverse events. We hypothesised that dexmedetomidine as an adjuvant would significantly prolong the duration of analgesia and improve perioperative outcomes without compromising motor function or safety.

## Materials and methods

This prospective, randomised, parallel-group comparative study was conducted in a tertiary care hospital in North India in accordance with the CONSORT guidelines between January 2024 and June 2024 after approval by the institutional ethics committee (GIMS/IEC/HR/2024/59) and prospective CTRI registration (CTRI/2024/12/078728). Written informed consent was obtained before enrollment. Sixty-seven patients were assessed for eligibility.

American Society of Anesthesiologists (ASA) physical status I-II patients, aged 18-60 years, posted for proximal femur fractures (fractures involving the neck of femur, pertrochanteric, or subtrochanteric regions) scheduled for surgery under spinal anaesthesia, with significant pain (numeric rating score (NRS) >4) and anticipated surgical duration <150 minutes, were included. The upper age limit was chosen to avoid confounding related to the institute’s geriatric anaesthesia protocol.

The sample size was calculated based on the hypothesis that the use of dexmedetomidine as an adjuvant in the PENG block in proximal femur fractures would significantly prolong the duration of analgesia. A pilot study (n=10, excluded from analysis) showed a mean duration of analgesia of 11.26±2.00 hours in Group A versus 7.46±2.43 hours in Group B. Using G*Power version 3.1.9.7, with a two-sided alpha of 0.05 and a power of 95%, a minimum of 22 patients per group was required. Thirty patients per group were enrolled to account for 20% anticipated attrition. 

Randomisation was performed using STATA 12 software (StataCorp LLC, College Station, Texas, USA) to generate the allocation sequence with permuted block sizes of 4, 6, and 8 in a 1:1 ratio. Sequentially numbered, sealed, opaque envelopes were used for allocation concealment and were opened by an independent anaesthesiologist not involved in the study. This anesthesiologist prepared identical 20.5 mL syringes labelled with the study number only. Participants, anesthesiologists, outcome assessors, and data analysts were blinded.

Patients were allocated to Group A (0.5 mL containing 50 mcg dexmedetomidine with 20 mL of 0.25% bupivacaine) or Group B (0.5 mL normal saline with 20 mL of 0.25% bupivacaine) for ultrasound-guided PENG block with spinal anaesthesia.

**Figure 1:**
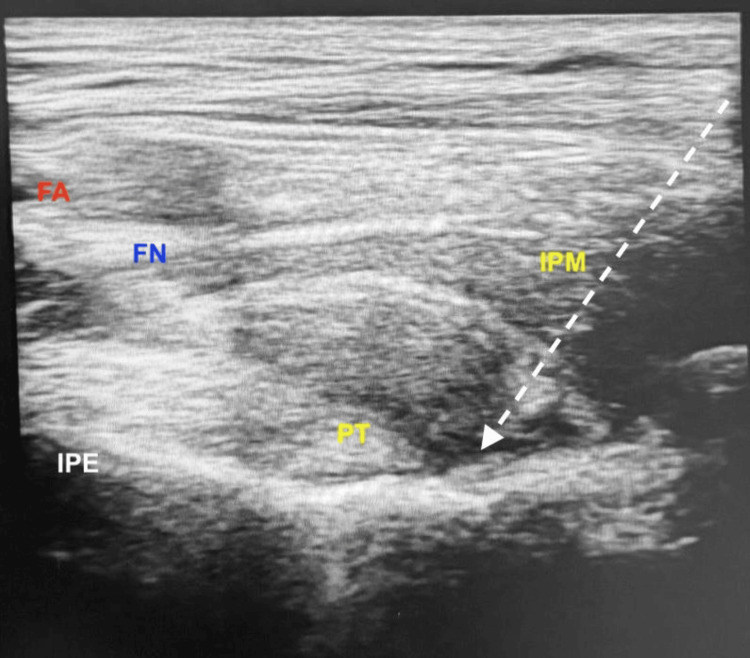
PENG block. White dotted line: needle trajectory; IPE: iliopubic eminence; PT: psoas major tendon; IPM: iliopsoas muscle; FA: femoral artery; FN: femoral nerve; PENG: pericapsular nerve group

After standard monitoring and establishment of intravenous access, the PENG block was performed by an independent experienced anesthesiologist not involved in the study, using a low-frequency curvilinear ultrasound probe (2-5 MHz) (FUJIFILM SonoSite Edge II, Bothell, WA, United States). The probe was placed over the anterior superior iliac spine and then slid toward the anterior inferior iliac spine with a 45-degree counterclockwise rotation to visualise the iliopsoas muscle and pubic ramus. Using a 23-G, 90 mm Quincke spinal needle, the study drug was injected using an in-plane approach (lateral to medial) into the fascial plane between the psoas tendon and pubic ramus after negative aspiration (Figure 1).

After 20 minutes of block placement, pain was reassessed and spinal anaesthesia was administered in the sitting position using a 26G Quincke spinal needle with 0.5% hyperbaric bupivacaine (12-15 mg) without adjuvants. The primary outcome was the duration of analgesia, defined as the time from block administration to the first request for rescue analgesia or an NRS score >4.

Secondary outcomes included ease of spinal positioning score, postoperative opioid requirement, quadriceps weakness at 24 hours, postoperative pain scores, and adverse effects, if any.

Ease of spinal positioning score was assessed using an investigator-defined four-point ordinal scale 20 minutes after the PENG block to evaluate patient comfort during positioning for spinal anaesthesia. Scores were defined as follows: 1, sitting comfortably without much help; 2, mild pain expressed verbally or by facial expressions; 3, severe pain but sitting position tolerated; and 4, unable to sit. All patients received standard intraoperative care and monitoring.

The first rescue analgesia was administered as tramadol 50-100 mg as per the institutional protocol. It was repeated after 30 minutes if the NRS remained greater than four, with the total dose not exceeding 300 mg in 24 hours. The total tramadol requirement in the first 24 hours was recorded.

Quadriceps weakness was assessed 24 hours postoperatively using the Oxford (Medical Research Council) grading scale ranging from 0 for no contraction to 5 for normal power [7].

Postoperative pain scores were assessed using the NRS at 0.5, 2, 4, 6, 12, and 24 hours. Adverse events like cardiovascular instability (bradycardia, hypotension, arrhythmias), respiratory complications, hematoma, local site infection, nerve injury, or local anaesthetic systemic toxicity were noted.

All patients received intravenous paracetamol 1 g before surgical closure, continued thrice daily postoperatively, and diclofenac 75 mg every 12 hours as part of multimodal analgesia. All patients were monitored for sedation using the Ramsay sedation score for the first 24 hours.

Data were entered into standard case record forms. Missing primary outcome data were planned to be handled using the last-observation-carried-forward method, and secondary outcomes with more than 5% missing data were to undergo multiple imputation. However, all study participants completed the 24-hour protocol with zero dropouts and complete data for all outcomes.

Selection, performance, detection, and attrition bias were minimised through concealed allocation, blinding, standardized protocols, and complete follow-up.

Data were analyzed using STATA 12. Normality of data was assessed using the Shapiro-Wilk test. Continuous variables were compared using the unpaired Student’s t-test or Mann-Whitney U test as appropriate. Categorical variables were analyzed using the chi-square or Fisher’s exact test. A p-value < 0.05 was considered statistically significant.

## Results

**Figure 2:**
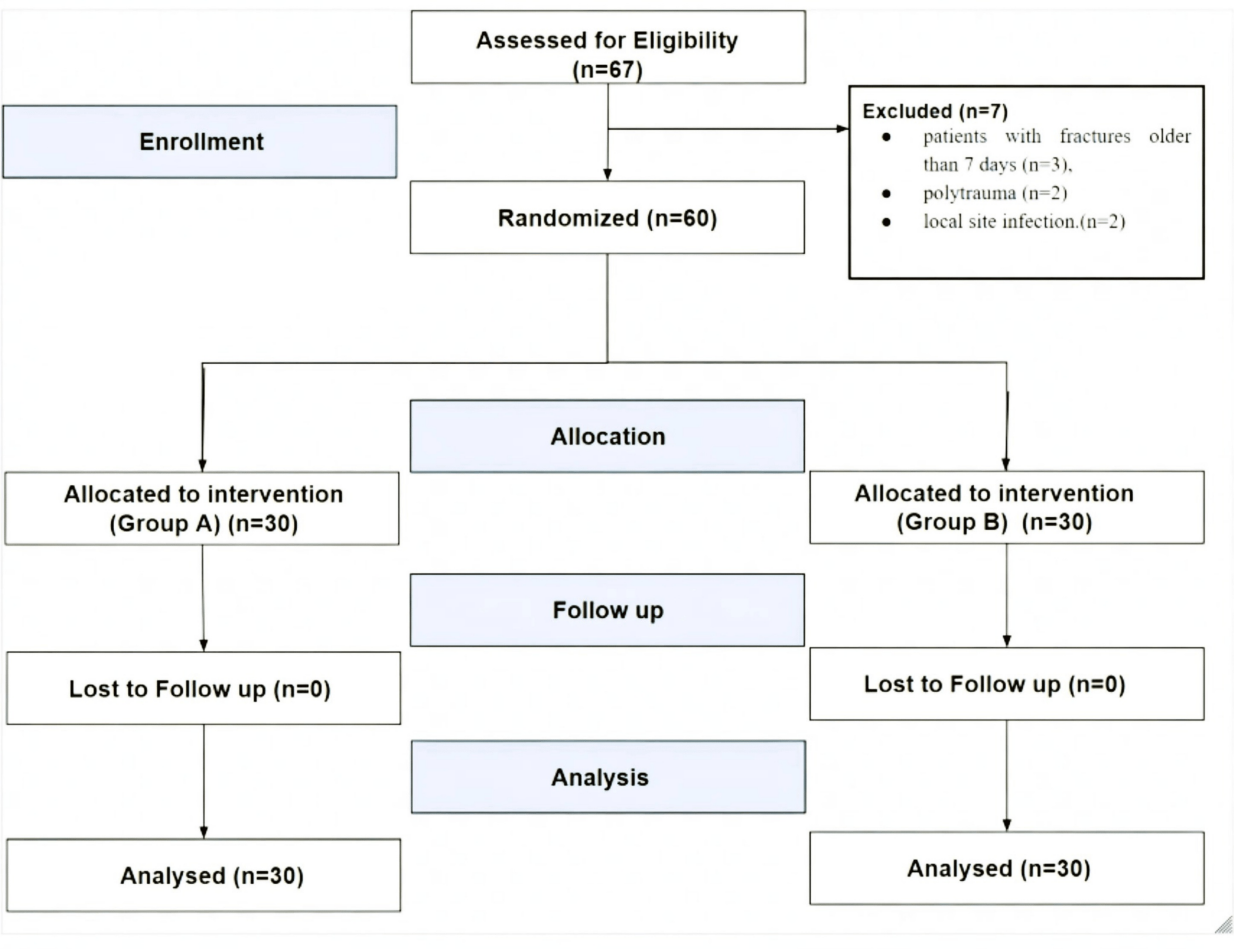
CONSORT flowchart CONSORT: Consolidated Standards for Reporting Trials

A total of 67 patients were assessed for enrollment in the study between January 2024 and June 2024, and 60 eligible participants were randomly allocated to Group A or Group B. All patients completed the 24-hour study protocol without any dropouts or protocol violations. All the exclusions occurred before randomisation; hence, selection bias was minimised (Figure 2).

The demographic characteristics, intraoperative hemodynamics, and baseline NRS were comparable between the groups (Table 1).

**Table 1:** Demographics, baseline characteristics and intraoperative hemodynamics §Normally distributed continuous variables: mean±SD, independent samples t-test (t-value). ||Categorical variables: n (%), Fisher's exact test. ‡Discrete ordinal variables (NRS): median [IQR], Mann-Whitney U test (U-value). *ASA: American Society of Anesthesiologists; †NRS: Numeric Rating Scale; ¶Measured after spinal anaesthesia. BMI: Body Mass Index; HR: Heart Rate; bpm: Beats per Minute; SBP: Systolic Blood Pressure; DBP: Diastolic Blood Pressure; MAP: Mean Arterial Pressure; SpO₂: Oxygen Saturation; IQR: Interquartile Range

Variable	Group A (n=30)	Group B (n=30)	Test Statistic	p
Demographics				
Age (years)§	47.7±11.2	46.3±10.6	t = 0.50	0.622
Weight (kg)§	62.4±7.1	64.1±8.2	t = -0.86	0.394
BMI (kg/m²)§	27.2±4.3	27.9±4.4	t = -0.64	0.526
ASA I/II*,||	15/15	22/8		0.111
Baseline vitals§				
HR (bpm)	80.3±8.2	83.6±5.9	t = -1.83	0.073
SBP (mmHg)	128.3±6.6	125.5±8.6	t = 1.39	0.171
DBP (mmHg)	80.4±7.0	81.7±7.6	t = -0.67	0.504
SpO₂ (%)	98.7±0.5	98.6±0.5	t = 0.27	0.791
Baseline NRS†,‡				
At rest	6 (6-6)	6 (6-7)	U = 390.0	0.305
On movement	7 (7-8)	7 (7-8)	U = 350.0	0.105
Intraoperative¶,§				
HR (bpm)	81.2±8.6	84.2±6.3	t = -1.54	0.129
SBP (mmHg)	124.2±6.8	123.3±7.6	t = 0.48	0.631
DBP (mmHg)	82.4±10.1	78.8±8.4	t = 1.49	0.142
MAP (mmHg)	96.3±7.7	93.7±8.0	t = 1.32	0.192
SpO₂ (%)	98.7±0.5	98.7±0.5	t = 0.00	1.000

**Figure 3:**
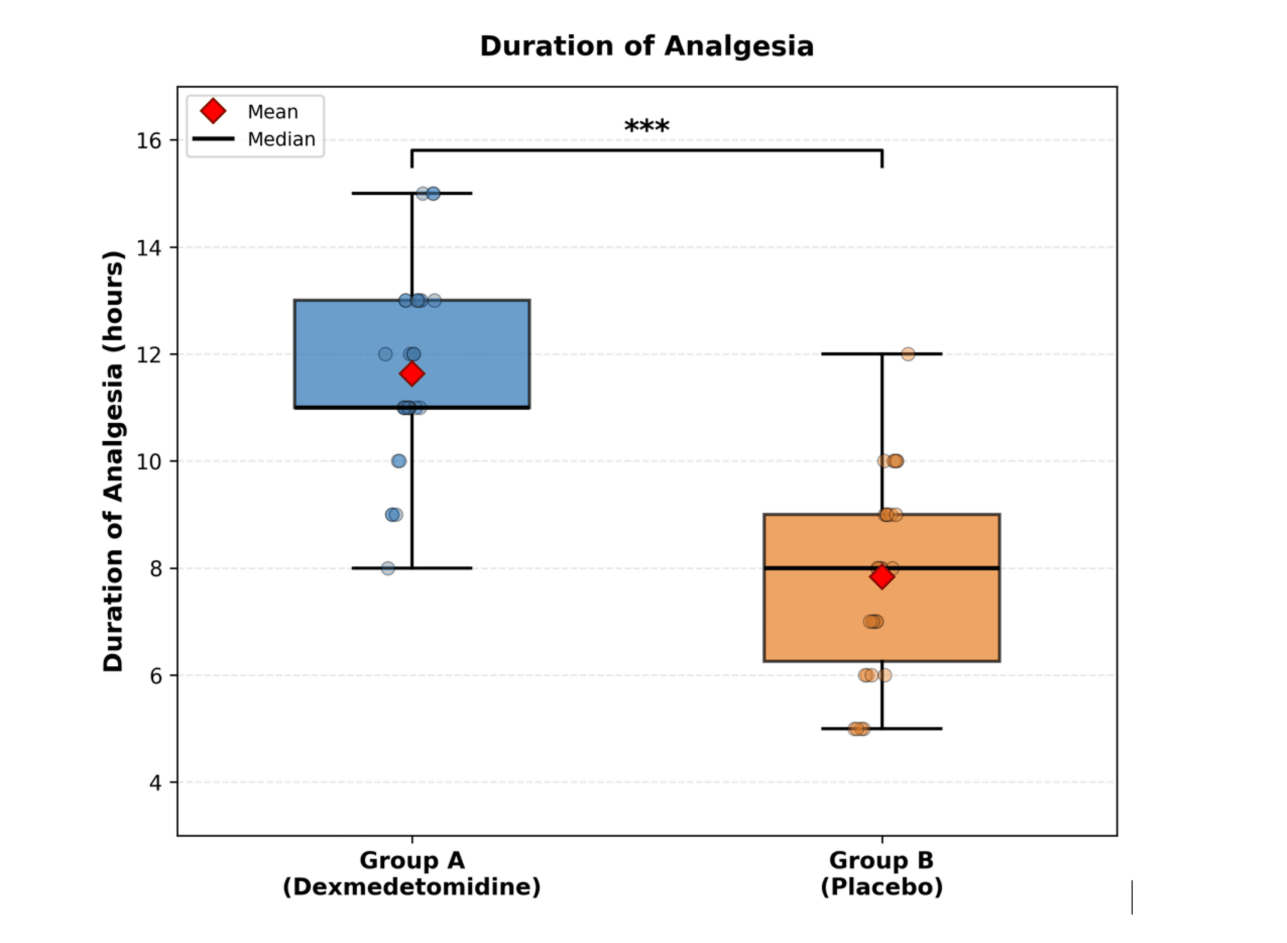
Duration of analgesia (time to first rescue analgesia) Box-and-whisker plots comparing the duration of analgesia (in hours) between Group A and Group B. Asterisks indicate statistical significance: ***p < 0.001, indicating a highly statistically significant difference (independent t-test).

The duration of analgesia was significantly prolonged in Group A (11.63±1.77 hours, 95% CI: 10.73-12.13) compared to Group B (7.83±1.84 hours, 95% CI: 7.13-8.40). There was a mean difference of 3.80 hours (95% 2.89-4.71; p < 0.001). The effect size was considerable, showing clinical benefit (large effect size, Cohen's d = 2.10) (Figure 3).

**Figure 4:**
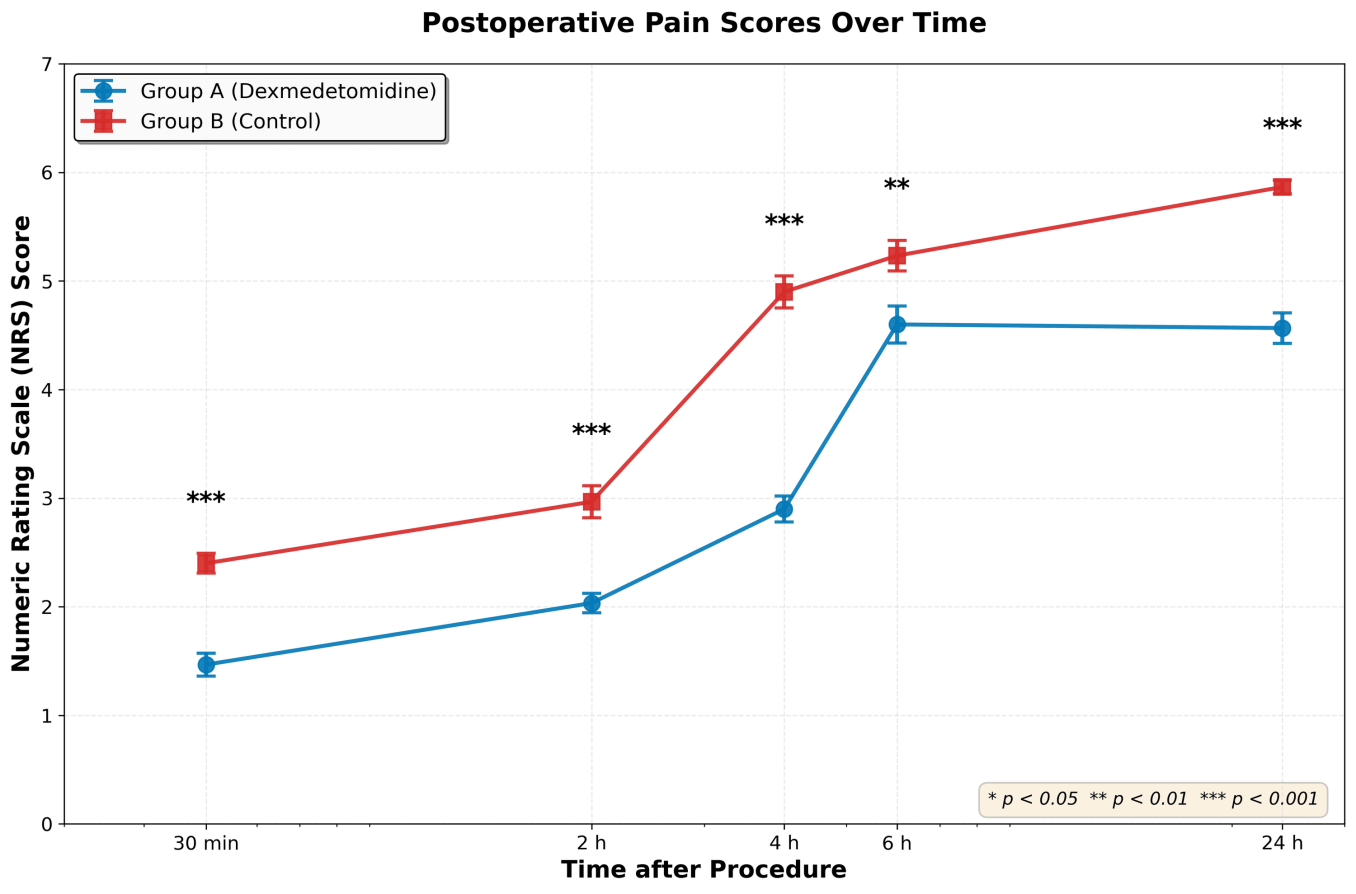
Postoperative pain scores Comparison of postoperative pain scores measured using the Numeric Rating Scale (NRS, 0-10). Data presented as mean ± standard error of the mean (SEM). Error bars represent SEM. Statistical comparisons were performed using the Mann-Whitney U test for each time point. Asterisks indicate statistical significance: * p < 0.05, ** p < 0.01, *** p < 0.001.

EOSP scores were significantly lower in Group A (p < 0.001). In Group A, 13 (43.3%) patients had a score of one, and the rest (56.7%) had a score of two. While in Group B, 27 (90%) patients had an EOSP score of two, and three patients (10%) even had an EOSP score of three. The postoperative NRS scores were significantly lower at all time points of data collection in Group A (Figure 4).

The 24-hour opioid consumption was statistically insignificant between the two groups (Table 2). Four patients in Group B required second rescue analgesia. Quadriceps muscle strength (Oxford score) showed no significant difference in both groups (p=0.702). No adverse events, like cardiovascular instability (bradycardia, hypotension, arrhythmias), respiratory complications, hematoma, local site infection, nerve injury, or local anaesthesia systemic toxicity, were noted (Table 2). Mild sedation (Ramsay score 2-3) was found in 16.7% of cases in Group A versus 3.3% of cases in Group B (p=0.14). No intervention was required for sedation in any case. 

**Table 2:** EOSP score, NRS at 20 minutes post procedure, postoperative opioid (tramadol) consumption, quadriceps score and adverse events Data presented as mean±SD, median (IQR), or n(%). *EOSP: Ease of Spinal Positioning Score (1-4 scale as defined); †NRS: Numeric Rating Scale (0-10); ‡Tramadol=rescue analgesic given as per protocol (50-100 mg IV, maximum dose-300 mg in 24 h), non-normally distributed (Shapiro-Wilk p<0.001); **Quadriceps muscle strength assessed at 24 hours using the Oxford grading scale (0=no contraction, 5=normal strength); Good strength=Oxford Grade 4-5, dichotomized as Good strength (Grade 4-5) vs Poor strength (Grade 0-3). P < 0.05 = statistically significant. The Mann-Whitney U test was used for NRS, EOSP, and tramadol. Categorical variables: n (%), chi-square test (χ²-value).

Outcome	Group A (n=30)	Group B (n=30)	Test Statistic	p
EOSP*,				
Median (IQR)	2 (1-2)	2 (2-2)	U = 229.5	<0.001
Score 1, n (%)	13 (43.3)	0 (0.0)	-	-
NRS at 20 min†,				
Median (IQR)	2(2-3)	3 (2-4)	U = 315.0	0.025
Tramadol‡ (mg)				
Median (IQR)	50 (50-100)	75 (50-100)	U = 360.0	0.119
Quadriceps strength**				
Good strength (Grade 4-5), n (%)	23 (76.7)	19 (63.3)	χ² = 0.71	0.398
Adverse events, n (%)	0 (0)	0 (0)	-	-

## Discussion

The current study showed that using dexmedetomidine as an adjuvant to bupivacaine for ultrasound-guided PENG block provides a significantly prolonged duration of analgesia, better EOSP scores, and lower postoperative pain scores without any notable side effects.

In this study, the dexmedetomidine group (Group A) showed a mean increase in the duration of analgesia by 3.80 hours (95% CI: 2.89-4.71; p < 0.001). Recent meta-analyses of dexmedetomidine as an adjuvant in peripheral nerve blocks have demonstrated an increase in the duration of analgesia with an earlier onset of sensory and motor block. This has been attributed to local axonal hyperpolarization and slowing of local anaesthetic washout due to vasoconstriction [8,9].

Dexamethasone has been used in various studies via the perineural route in the PENG block, resulting in a prolonged duration of analgesia and an opioid-sparing effect in the postoperative period [10,11]. However, robust studies using perineural administration of dexmedetomidine in the PENG block are lacking.

A recent systematic review of the optimal dosage of perineural dexmedetomidine has shown that it prolongs the duration of analgesia by a mean period of five hours after brachial plexus blockade with a dose range of 30-50 mcg. Doses greater than 60 mcg result in an increased incidence of adverse cardiovascular events such as hypotension and bradycardia. Hence, a 50 mcg dose of dexmedetomidine was used in the present study, with no significant adverse effects and a similar clinical impact in terms of prolonging the duration of analgesia [12].

Difficulty in positioning the patient for neuraxial anaesthesia is another challenge in patients with proximal femur fractures. With the use of dexmedetomidine in the PENG block, an EOSP score of one (sitting without pain and minimal help) was achieved in 43.3% of the patients (P < 0.001). This can be attributed to observations from several studies, which report an earlier onset with the use of perineural dexmedetomidine in combination with local anaesthetics in peripheral nerve blocks [13]. Similarly, femoral and fascia iliaca blocks with dexmedetomidine as an adjuvant have shown improved analgesia for positioning for spinal anaesthesia [14].

Lower NRS scores were observed in the dexmedetomidine group at all time points, 20 minutes after block administration and up to 24 hours postoperatively. This sustained reduction in postoperative pain scores facilitates early mobilization, better participation in physical therapy, and enhanced recovery after hip surgeries [9,15].

The main advantage of using the PENG block is its motor-sparing analgesic effect. Use of dexmedetomidine does not affect motor function, as observed in the present study, as quadriceps strength was preserved in both groups. Literature suggests that the impact on motor function depends mainly on the local anaesthetic type, concentration, and volume used for the peripheral nerve block. Recent studies have reported lower motor weakness with lower volumes (10-20 ml) in the PENG block compared to larger volumes (30 ml), with postoperative quadriceps weakness rates up to 75% [16]. Larger volumes propagate to the femoral nerve or iliacus compartment, resulting in volume-dependent motor blockade [16,17]. Hence, for the PENG block, 20 ml of 0.25% bupivacaine with 50 mcg of dexmedetomidine was used for an optimal clinical outcome. No adverse cardiovascular events were noted. Low doses of perineural dexmedetomidine have been well tolerated in most studies [15,18].

However, this study has certain limitations. First, the mild sedative effect of dexmedetomidine may influence the subjective assessment of pain and EOSP. Additionally, serum concentrations of dexmedetomidine were not measured; therefore, the contribution of systemic absorption cannot be ruled out. Second, EOSP was assessed using an investigator-defined scale, which may introduce measurement bias. Third, although baseline characteristics were comparable between the two groups, factors such as fracture subtype, variation in surgical technique, and individual pain perception may have acted as potential confounders.

Proximal femur fractures are most common in the elderly population. As this was a single-centre trial and geriatric patients aged>60 years were excluded in accordance with institutional protocol, the external validity of the study is limited. Lastly, a fixed dose of dexmedetomidine was used; optimal dosing requires further investigation, and objective assessment of muscle strength is warranted.

## Conclusions

In this single-centre trial, the use of dexmedetomidine as a perineural adjuvant in the PENG block was associated with a prolonged duration of analgesia (95% CI: 2.89-4.71, p < 0.001), better positioning scores (p < 0.001), and lower postoperative pain compared to bupivacaine alone in 60 patients aged 18-60 years. Motor function was preserved in both groups, with no significant adverse events. However, the clinical applicability of the study is limited to the younger adult population (ages 18-60 years). Further large-scale, multicentre studies including geriatric patients, with objective functional assessments and dose-response evaluation, are needed to evaluate the clinical efficacy and safety of dexmedetomidine as a perineural adjuvant in the PENG block.
